# Real-world adherence trajectories to direct oral anticoagulants in naive patients with atrial fibrillation in Spain

**DOI:** 10.3389/fphar.2025.1562620

**Published:** 2025-07-31

**Authors:** L. M. Leguízamo-Martínez, M. E. Ballarin, I. Hurtado, G. Sanfélix-Gimeno, F. Sánchez-Sáez, M. Sabaté

**Affiliations:** 1 Department of Clinical Pharmacology, Area Medicament, Hospital Clinic of Barcelona, Barcelona, Spain; 2 Department of Clinical Pharmacology, Institut d’Investigacions Biomèdiques August Pi i Sunyer (IDIBAPS), Barcelona, Spain; 3 Department of Pharmacology, Therapeutics and Toxicology, Universitat Autònoma de Barcelona (UAB), Barcelona, Spain; 4 Clinical Pharmacology Service, Vall d’Hebron Hospital Universitari, Vall Hebron Institut de Recerca (VHIR), Barcelona, Spain; 5 Health Services Research and Pharmacoepidemiology Unit, Fundacio per al Foment de la Investigacio Sanitaria i Biomedica (FISABIO), Valencia, Spain; 6 Red de Investigación en Cronicidad, Atención Primaria y Prevención y Promoción de la Salud - RICAPPS, Barcelona, Spain; 7 Department of Applied Statistics and Operational Research, and Quality, Universitat Politècnica de València, Valencia, Spain

**Keywords:** direct oral anticoagulants (DOACs), adherence trajectories, atrial fibrillation (AF), group-based trajectory modeling (GBTM), real-world data analysis

## Abstract

**Aims:**

This study aimed to identify adherence trajectories and associated factors in atrial fibrillation patients who initiated direct oral anticoagulant (DOAC) therapy, using population-based data from the Catalonia and Valencia regions in Spain during a 2-year follow-up.

**Methods and Results:**

Group-based trajectory modelling (GBTM) was applied to assess adherence patterns in cohorts comprising 14,641 patients in Catalonia and 13,211 in Valencia. Adherence trajectories were categorised based on prescription data, revealing three main trajectories in Valencia and five in Catalonia. Most patients in Valencia demonstrated high adherence, whereas Catalonia showed more varied patterns, including early, gradual, and late declines. Factors associated with non-adherence included high co-insurance levels, alcohol use, and specific DOACs, such as dabigatran.

**Conclusion:**

Adherence trajectories differed between the two regions, with three identified in Valencia and five in Catalonia. Shared non-adherence patterns were observed across both cohorts, while Catalonia exhibited additional noncompliance trends. Key factors associated with non-adherence included socio-economic variables, clinical characteristics, and the type of DOAC prescribed. Understanding these patterns is essential for developing targeted intervention strategies to improve adherence and optimise treatment outcomes.

## 1 Introduction

Atrial fibrillation (AF) is the most common sustained heart rhythm disorder worldwide, with a great impact on public health due to the increased risk of stroke and hospitalisations. The currently estimated prevalence of AF in adults is between 2% and 4% (Benjamin et al., 2019). Previously, the main AF treatments were vitamin K antagonists (VKA) such as warfarin and acenocoumarol, which required regular monitoring of the International Normalised Ratio (INR) and dietary restrictions. In the 2024 ESC guidelines, the direct oral anticoagulants (DOACs), dabigatran, rivaroxaban, apixaban, and edoxaban, are recommended over VKA for the prevention of stroke and systemic embolism in patients with AF, except in those with moderate to severe mitral stenosis or a mechanical heart valve and are associated with a lower risk of major bleeding (Van Gelder et al., 2024). Multiple studies have shown that non-adherence to DOACs in patients with AF may increase the risk of adverse outcomes (Géric et al., 2017; Sherwood et al., 2014; Reilly et al., 2014; Alberts et al., 2016).

Several observational studies using pharmacy claims databases or electronic health records (EHR) have been conducted to estimate the real-world use of oral anticoagulants (OACs), both DOACs and VKA (Hurtado-Navarro et al., 2018; Giner-Soriano et al., 2020; Giner-Soriano et al., 2023). A meta-analysis of 30 observational studies related to the measurement of adherence to OACs, its determinants and impacts on patients with AF, found that the pooled proportion of adherent patients at 6 months and 1 year were 63% and 70%, respectively. Despite these figures, up to 30% of patients were non-adherent to OACs. Non-adherent patients were more likely to experience stroke and death compared with adherent patients (Salmasi et al., 2020). A systematic review of secondary adherence to DOACs described that several articles have shown adherence figures to DOACs in patients with non-valvular atrial fibrillation (NVAF) greater than 80% or 90% for the proportion of days covered (PDC) or the rate of medication possession (MPR). In addition, the proportion of patients with PDC80 or MPR80 was above 70%–75%. These figures are higher than current estimates for other drugs for chronic diseases (Rodríguez-Bernal et al., 2018).

Medication adherence is often estimated from EHR using prescription and dispensing databases. Numerous variations of the MPR or PDC are commonly reported as aggregate or “point” estimates of medication availability for an individual over a given observation period (Dima et al., 2017). However, thresholds to differentiate adherence from non-adherence typically refer to an aggregated value over the entire observation period, disregarding differences in adherence over time (Allemann et al., 2019). Therefore, it is important to consider the entire period of adherence, which can be achieved, for example, by using the Group Trajectory Modelling (GBTM) approach.

GBTM is a methodological approach that visually describes the dynamics of medication adherence and classifies adherence behaviour (Alhazami et al., 2020). It is a semiparametric model for longitudinal data that postulates a discrete distribution of the population, enabling the identification of subgroups/classes of individuals with homogeneous trajectories within the population (Nguena Nguefack et al., 2020). A systematic literature review suggests that adherence trajectories and predictors of specific group membership may be consistent across diverse disease states (Alhazami et al., 2020).

Among the existing literature, five studies have investigated adherence trajectories to OAC or DOAC. Three of these studies focused on short-term adherence trajectories (1 year) after an AF diagnosis (Hernandez et al., 2019; Chen et al., 2020; Salmasi et al., 2021), while two studies examined long-term adherence trajectories over periods of 3.5 and 5 years (An et al., 2021; Mohan et al., 2022). These studies reported between 3 and 4 different adherence trajectories, with the proportion of adherent patients ranging from 40% to 85%. Regarding factors associated with adherence, one study reported that clinical and demographic characteristics are inadequate to predict the adherence trajectories of patients (Salmasi et al., 2021). However, another study reported that predictors such as lower CHA_2_DS_2_-VASc (0–1 versus ≥5) and previous injury falls were associated with both early discontinuation and gradually declining adherence trajectories (An et al., 2021).

However, there is scant information in the medical literature regarding longitudinal real-world data on medication adherence to DOACs in the AF population, and current publications focus on the North American population. In addition, there is heterogeneity between the published results regarding adherence patterns and associated factors.

In this study, the objectives were to characterise adherence patterns in new users of DOACs with AF and to identify factors associated with non-adherence, using real-world data from two populations in Spain.

## 2 Methods

### 2.1 Design

We conducted a retrospective cohort study to identify new users of oral anticoagulants (OAC) among patients with atrial fibrillation (AF) in two regions of Spain: Catalonia and Valencia. The study included patients who were newly prescribed a direct oral anticoagulant (DOAC) between 1 January 2012, and 31 December 2018, in Catalonia, and between 1 January 2012, and 31 December 2017, in Valencia (Supplementary Figure S1). Patients were followed for up to 2 years from the date of their first DOAC prescription (index date). As part of a sensitivity analysis, the study was repeated with a 1-year follow-up period.

### 2.2 Population and setting

The study was conducted in the population covered by the Catalan Health System (CatSalut) and Valencian Health System (VHS), which are part of the Spanish National Health System (sNHS). We included patients aged 18 years or older with a diagnosis of AF or atrial flutter (International Classification of Diseases, Ninth Revision, Clinical Modification [ICD-9-CM] codes of 427.3x; International Classification of Diseases, 10th Revision, Clinical Modification [ICD-10-CM] codes of I48.x) who started treatment with DOAC (dabigatran, apixaban, edoxaban, or rivaroxaban) for the prevention of thromboembolic events during the recruitment period in each population. A 1-year look-back period was used to define baseline patient characteristics and to exclude prevalent OAC users. While we acknowledge that a longer look-back might reduce the risk of misclassifying prevalent users as new users, we considered 1 year to be a reasonable balance between minimizing misclassification and retaining a sufficient sample size. We also excluded patients with concomitant diagnoses related to mitral valve disease or mechanical heart valve and indication for DOAC due to hip or knee arthroplasty (Codes in Supplementary Table S1). Patients with registry errors (death date before the prescription date and prescription of two or more different DOACs) and patients with limitations of follow-up (non-residents or non-pharmaceutical coverage) were also excluded.

### 2.3 Data sources

Data were obtained from the Data Analytic Program for Health Research and Innovation (PADRIS) in Catalonia and the Valencia Health System Integrated Database (VID) in Valencia. These two data sources cover almost one-third of Spain’s population, approximately 28%.

PADRIS obtains data from CatSalut’s population-based electronic information systems, a universal healthcare system in Catalonia for its approximately 9 million inhabitants. It is a public program led and managed by the Catalan Agency for Quality and Assessment of Health (AQuAS) of the Department of Health, which promotes research and innovation to improve the health of citizens, following the guidelines designed by the European Commission (Government of Catalonia, 2024).

VID integrates data sources of the population covered by the Valencian Health System, which serves approximately 5.3 million inhabitants in the Valencia region. This information comes from multiple, public, population-wide electronic databases, which combine data sources linking them at an individual level through a single anonymised patient identifier (García-Sempere et al., 2020).

The data provided includes the EHR from all primary care healthcare centres, medical prescriptions, dispensing from community pharmacies and death registration. Hospitalisation information includes clinical and administrative data on all hospital discharges based on the Minimum Basic Data Set (MBDS) at hospital discharges.

### 2.4 Covariates

Demographic and clinical characteristics were included using a 1-year look-back period. Demographics included age and sex. Clinical characteristics included comorbidities (Supplementary Table S1), and CHA_2_DS_2_-VASc and HAS-BLED scores to assess stroke and bleeding risk. Specific concomitant medications dispensed in the 3 months prior to the index date were collected including antiplatelet agents, antiarrhythmic agents, non-steroidal anti-inflammatory drugs (NSAIDs) and COXIBs. Polypharmacy was considered the prescription of 5 or more drugs before the index date (Masnoon et al., 2017). Coinsurance (the percentage of the drug price paid by the patient) was categorised into low (0%–10%) or high (40%–60%) coinsurance.

### 2.5 Adherence measures

The primary measure of secondary adherence was adherence trajectories over time for 2-year of follow-up from the index date. These were estimated with the “lcmm” package of R (Yeaw et al., 2009; Proust-Lima et al., 2017; Proust-Lima et al., 2022), which fits latent class mixed models for different types of outcomes, such as continuous longitudinal outcomes or discrete outcomes. First, days’ supply with DOAC prescription and dispensing were ascertained in each cohort. Consecutive 30-day windows were created for each patient from the date of their first DOAC prescription until switching to another OAC or the end of follow-up (1 and 2 years). Patients who switched were censored at the time of switching and therefore removed from both the numerator and the denominator in adherence calculations from that point onward. In cases of oversupply, the stockpiling was considered with a maximum supply of 90 accumulated days (Hurtado-Navarro et al., 2018; Salmasi et al., 2020). PDC was calculated for each 30-day interval as the percentage of days covered by filled prescriptions, using the total number of days prescribed as the denominator. Two different PDC estimations were used as outcomes: (1) Continuous PDC (values between 0% and 100%); and (2) Dichotomous/binary PDC (using a threshold of 80%; PDC≥80%: 1, PDC<80%: 0).

A grid of parameters was used to determine the best trajectory model, testing different polynomial degrees (2, 3, and 4) and numbers of classes (2–5). The final model was selected using an adaptation of the Nagin criteria (Nagin and Odgers, 2010; Nagin, 2014). Based on Nagin’s approach, we applied several criteria to identify the optimal number of trajectories. First, each trajectory class was required to include at least 5% of the individuals to ensure the trajectories were representative of the study population. Second, we set an entropy threshold of greater than 75% to ensure a high level of certainty in classifying individuals into different trajectories. Finally, the Bayesian Information Criterion (BIC) was used as the primary selection criterion. The model with the lowest BIC, subject to these constraints, was selected as the optimal model for describing the data.

Additionally, PDC was estimated for the whole follow-up. The threshold above 80% was considered the cut-off point to classify patients as adherent, a threshold above which patients can be considered highly adherent for most classes of chronic medications (Nagin, 2014).

### 2.6 Statistical analysis

First, both cohorts were selected by applying the inclusion and exclusion criteria. Descriptive statistics were used to describe the demographic and clinical characteristics of the patients of the two cohorts. Second, DOAC adherence trajectories were calculated, and the best trajectory model was selected for each cohort based on previously specified parameters. The probability of being adherent for each trajectory class/group was plotted over time, and the PDC for the whole follow-up for each trajectory was calculated. Additionally, PDC for the whole follow-up was estimated for both cohorts (at 1-and 2-year) and was described as a continuous (mean [95% Confidence interval]) and a binary measure (% [95% Confidence interval]). Finally, the factors associated with each adherence trajectory were evaluated for each cohort and follow-up (1-and 2-year). Multinomial logistic regression models were used to assess the association between baseline characteristics and membership with each identified adherence trajectory. The class with full adherence to DOAC therapy was treated as the reference group. Odds ratios (ORs) and 95% CIs comparing the other classes to the reference group were calculated. The reference values used were male for sex, age greater than or equal to 65 years for age, apixaban for the active principle and low co-insurance. All statistical analyses were performed using R 4.2.2.

### 2.7 Ethics approval

The Drug Research Ethics Committee of the Hospital Clínic of Barcelona approved the study protocol on 22 September 2022. Retrospective and pseudonymised data were transferred to the research team in compliance with Spanish law and EU regulations into account.

### 2.8 STROBE guidelines compliance

This manuscript follows the Strengthening the Reporting of Observational Studies in Epidemiology (STROBE) guidelines to ensure transparency and quality in reporting. The completed STROBE checklist is provided as a Supplementary Material.

### 2.9 Data availability statement

The data used and analysed during the current study are available from the Catalonia and Valencia region governments upon reasonable request. Data sharing is subject to ethical approval and institutional policies to protect patient confidentiality and comply with data protection regulations.

### 2.10 Missing data

There was no missing data recorded for the included variables in our study, as it utilised retrospective electronic health records (EHR) with complete documentation. However, it is acknowledged that data for some individuals might not be captured due to potential errors in the recording process or other unforeseen factors. These instances are inherent limitations of EHR-based studies.

## 3 Results

**FIGURE 1:**
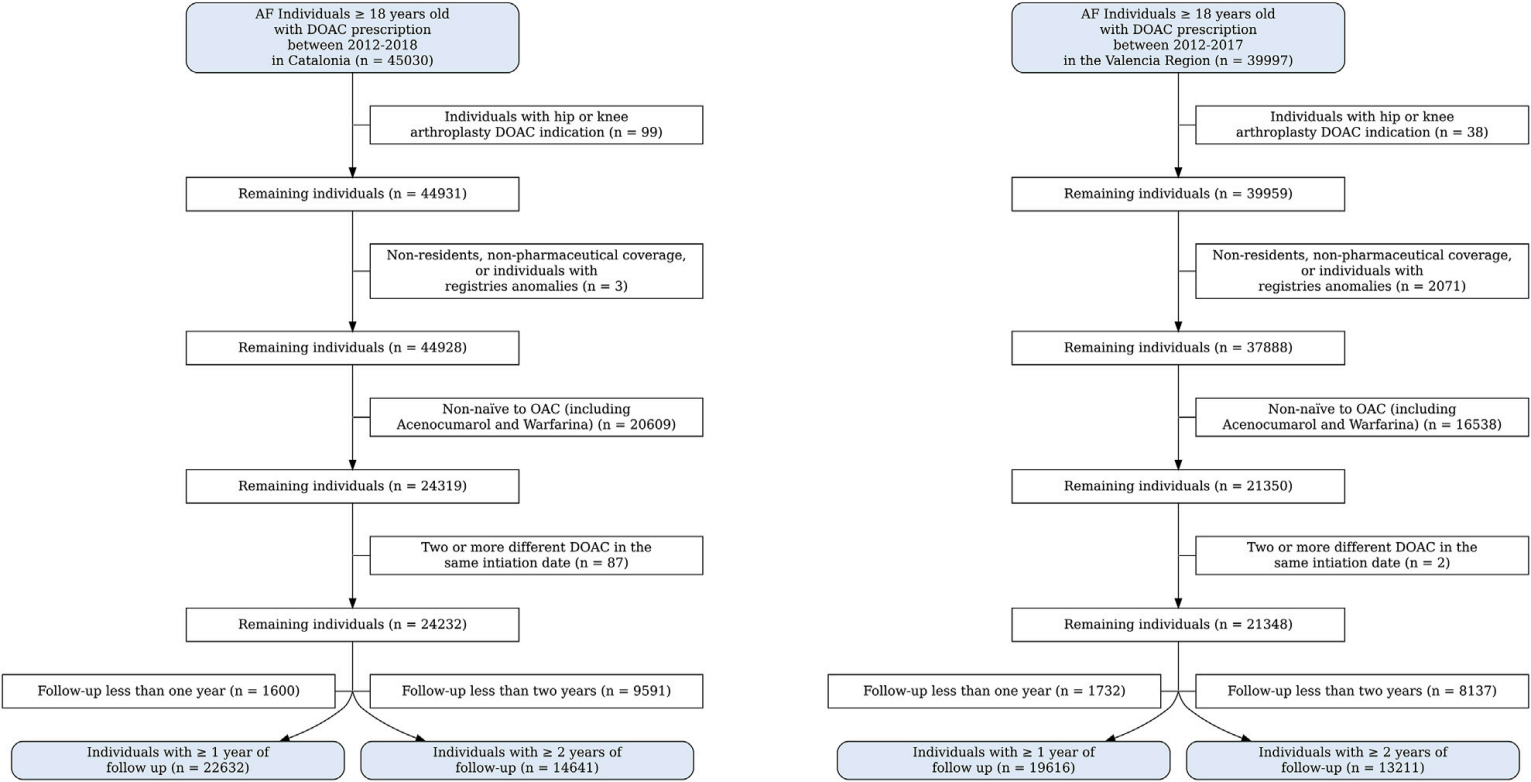
Flowcharts of catalonia and valencia region.

The study cohorts consisted of 14,641 patients with AF initiating DOAC therapy and OAC-naïve with 2-year of follow-up in Catalonia, and 13,211 patients in the Valencian region (Figure 1; Supplementary Figure S1). The age group with the highest percentage of patients was 75–84 years, and the most frequently used DOACs were rivaroxaban and apixaban in both cohorts. In Catalonia, patients were more likely to be women (55.5%) while in Valencia, patients were more likely to be men (55.1%). Hypertension (C: 67.3%; V: 74.7%), diabetes (C: 24.1%, V: 25.9%) and vascular disease (C: 18.6%, V: 22.4%) were the most common comorbidities. The mean CHA_2_DS_2_-VASc score was above 2 (high risk of ischaemic stroke) in both cohorts, and approximately half of the patients had a HAS-BLED score ≥3, which is considered high bleeding risk: 47.8% in the Catalonia cohort and 53.2% in the Valencia cohort. In both cohorts, over 50% of the patients were prescribed more than five concomitant medications and over 80% of the patients had a low level of co-insurance (Table 1).

**TABLE 1:** Population baseline characteristics at 2-year follow-up.

Variable	Category	Valencia (n = 13,211)	Catalonia (n = 14,641)
Active Principle, n (%)	Apixaban	4,135 (31.3%)	4,755 (32.5%)
	Dabigatran	4,254 (32.2%)	3,859 (26.4%)
	Edoxaban	141 (1.1%)	769 (5.3%)
	Rivaroxaban	4,681 (35.4%)	5,258 (35.9%)
Age, n (%)	<65	3,095 (23.4%)	3,508 (24.0%)
	65–74	3,995 (30.2%)	4,116 (28.1%)
	75–84	4,413 (33.4%)	4,692 (32.0%)
	≥85	1,708 (12.9%)	2,325 (15.9%)
	mean (sd)	72.42 (11.75)	72.79 (12.25)
Sex, n (%)	Man	7,278 (55.1%)	6,508 (44.5%)
	Woman	5,933 (44.9%)	8,133 (55.5%)
Comorbidities, mean (sd)	Alcohol	377 (2.9%)	699 (4.8%)
	Congestive heart failure	2,345 (17.8%)	1,665 (11.4%)
	COPD	715 (5.4%)	1,429 (9.8%)
	Dementia	854 (6.5%)	714 (4.9%)
	Depression	1,796 (13.6%)	2,080 (14.2%)
	Diabetes	3,423 (25.9%)	3,526 (24.1%)
	Gastrointestinal bleeding	594 (4.5%)	808 (5.5%)
	Hypertension	9,875 (74.7%)	9,851 (67.3%)
	Intracranial Haemorrhage	257 (1.9%)	233 (1.6%)
	Ischemic stroke	1,588 (12.0%)	1,550 (10.6%)
	Liver disease	434 (3.3%)	131 (0.9%)
	Malignancy	1,530 (11.6%)	2,473 (16.9%)
	Other bleeding	1,718 (13.0%)	1,271 (8.7%)
	Renal disease	1,477 (11.2%)	1,841 (12.6%)
	TIA	599 (4.5%)	702 (4.8%)
	Vascular disease	2,956 (22.4%)	2,728 (18.6%)
	VTE	954 (7.2%)	791 (5.4%)
Scores, mean (sd)	CHADS_2_	1.96 (1.35)	1.81 (1.34)
	CHA_2_DS_2_-VASc	3.40 (1.84)	2.75 (1.68)
	HAS-BLED	2.58 (1.24)	2.43 (1.28)
CHA_2_DS_2_-VASc Categories, n (%)	0–1	2,151 (16.3%)	3,581 (24.5%)
	2	2,156 (16.3%)	2,943 (20.1%)
	3	2,612 (19.8%)	3,538 (24.2%)
	4	2,661 (20.1%)	2,292 (15.7%)
	≥5	3,631 (27.5%)	2,287 (15.6%)
HAS-BLED Categories, n (%)	0–1	2,522 (19.1%)	3,450 (23.6%)
	2	3,652 (27.6%)	4,192 (28.6%)
	3	4,140 (31.3%)	4,090 (27.9%)
	≥4	2,897 (21.9%)	2,909 (19.9%)
Treatment in the 3 Months Previous to the Index Date, n (%)	APT	6,119 (46.3%)	6,401 (43.7%)
	NSAID	1,644 (12.4%)	1,832 (12.5%)
	Coxib	558 (4.2%)	175 (1.2%)
	anti-arrhythmic	2,624 (19.9%)	2,375 (16.2%)
Polypharmacy, n (%)	mean (sd)	7.68 (3.97)	5.19 (3.02)
	≥5	10,324 (78.1%)	7,961 (54.4%)
Coinsurance*	low (0%–10%)	10,818 (81.9%)	12,722 (86.9%)
	high (40%–60%)	2,393 (18.1%)	1,919 (13.1%)

*Coinsurance is expressed as a percentage of the total cost of prescriptions to be paid. AF, atrial Fibrillation; COPD, chronic obstructive pulmonary disease; VTE, venous thromboembolism; APT, antiplatelet therapy; NSAID, Non-steroidal anti-inflammatory drugs.

**FIGURE 2:**
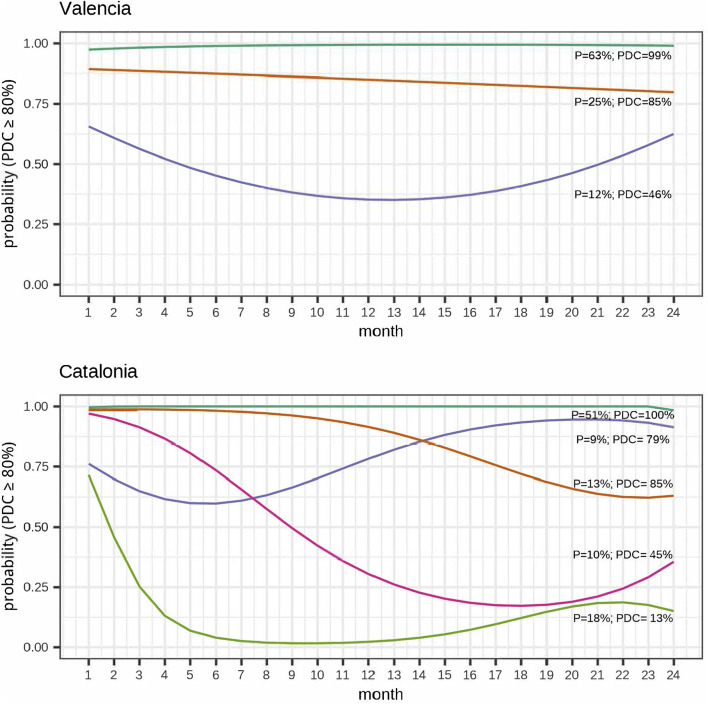
Adherence trajectories at 2-year follow-up (Binary adherence).

The optimal trajectory model was identified based on the criteria defined above for each cohort, period, and type of outcome (continuous or dichotomous PDC measure). Different adherence patterns were observed depending on the region. We chose the binary model because it was more informative, enabling an additional trajectory in the Catalonia cohort and allowing for a finer classification of the adherence patterns. This enabled us to capture distinct patterns of adherence and improved the accuracy of our analysis (Figure 2).

In Catalonia five adherence trajectories were identified at 2 years of follow-up: fully adherent patients (“fully adherent” 7,409[51%]); patients with an early decline to non-adherence (“early decline” 2,607[18%]); patients with a late drop to non-adherent (“late decline” 1,832[13%]); patients who gradually declined to non-adherent (“gradual decline” 1,435[10%]), and patients who initially declined to non-adherence and subsequently regained adherence (“gap and recovery” 1,358[9%]). In the Valencia region, three adherence trajectories were identified: “fully adherent” (8,312[63%]), highly adherent patients (“highly adherent” 3,261[25%]) and gap and recovery (1,638[12%]).

Regarding secondary adherence, the mean PDC was 79% (95%CI: 78%; 79%) and 92% (95%CI: 91%; 92%) for the Catalonia and the Valencia cohorts, respectively. While the percentage of adherent patients (PDC≥80%) was 69% (68%; 69%) for Catalonia and 87% (86%; 88%) for the Valencia region. PDC for the whole follow-up for each trajectory group is presented in Figure 2. Additionally, adherence trajectories for 1-year follow-up (binary PDC) and trajectories at 1- and 2-year follow-up for continuous PDC are shown in Supplementary Figures S2–S4.

### 3.1 Factors associated with adherence trajectories

**FIGURE 3:**
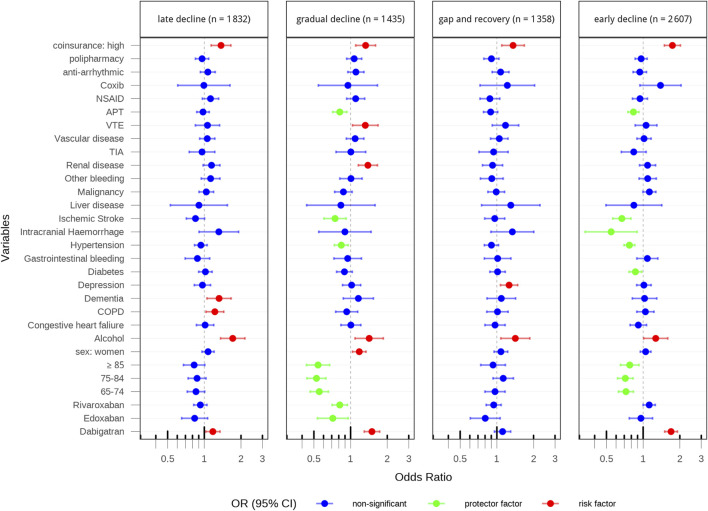
Factors associated with adherence trajectories at 2-year follow-up in catalonia.

**FIGURE 4:**
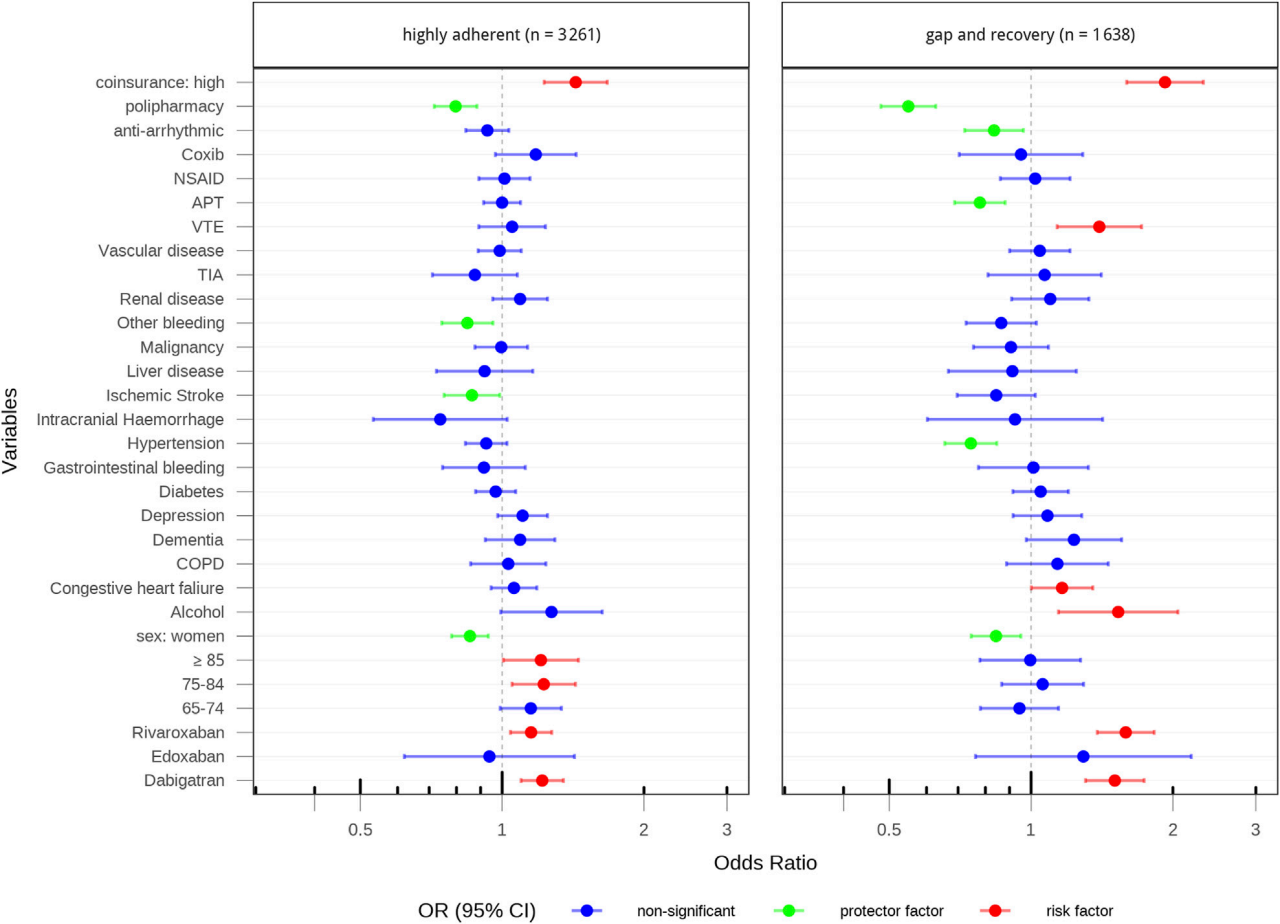
Factors associated with adherence trajectories at 2-year follow-up in valencia.

Figures 3, 4 show the factors associated with adherence trajectories at 2-year follow-up for Catalonia and Valencia, respectively. In both cohorts, factors such as higher co-insurance and alcohol consumption were consistently associated with increased odds of being in a poor adherence trajectory, while patients with previous ischemic stroke, hypertension or previous treatment with antiplatelets were less likely to show poor adherence behaviours. Patients initiating with dabigatran or rivaroxaban, depending on the cohort, compared to those initiating with apixaban, were more likely to show poor adherence behaviours. These patterns largely mirrored those seen at the 1-year follow-up where the study cohorts consisted of 22,632 patients with AF initiating DOAC therapy and OAC-naive in Catalonia and 19,616 patients in the Valencian region (Supplementary Table S2; Supplementary Figures S5, S6).

## 4 Discussion

In this study, we applied GBTM to two population-based cohorts (from the Catalonia and Valencia regions, covering a large population of approximately14 million inhabitants of the Spanish territory), comprising patients with AF who initiated treatment with a direct oral anticoagulant, to assess their adherence during 2 years of follow-up. We identified five and three adherence trajectories at 2 years of follow-up in the Catalonia and Valencia cohorts, respectively. The Catalonia cohort showed greater heterogeneity and a higher proportion of patients with poor adherence (28%) compared to Valencia (12%). Factors consistently associated with poor adherence in both regions included high co-insurance levels and alcohol consumption, while high-risk patients (with previous ischemic stroke or hypertension or previous treatment with antiplatelets) were less likely to show poor adherence behaviours. Additionally, initiating treatment with dabigatran or rivaroxaban, compared to apixaban, was associated with a higher likelihood of poor adherence. These results were consistent across sensitivity analyses.

Several previous studies have explored oral anticoagulant adherence trajectories among atrial fibrillation patients using group-based trajectory models. For example, Salmasi et al. (2021), using data from Population Data British Columbia from 1996 to 2019, identified four trajectories at 5 years of follow-up: consistent adherence (74%), rapid decline and discontinuation (12%), rapid decline with partial recovery (10%), and slow decline and discontinuation (4%). In another study of patients initiating DOAC, using Kaiser Permanente Southern California data from 2012 to 2018, three groups of trajectories were identified at 2 years: consistent adherence (79.2%), early discontinuation (12.9%), and gradual decline (7.9%) (An et al., 2021). A study using Medicare claims data (2013–2016) found four trajectories at 1 year (including non-treated patients in a newly diagnosed AF population): never used OAC (43.8%), late initiators (7.6%), early discontinuers (8.9%), and continuously adherent (40.1%) (Hernandez et al., 2019). While these studies offer valuable insights, their findings are not directly comparable to those of the present study due to differences in populations, healthcare systems, follow-up periods, and data sources. Despite these methodological differences, our results are consistent with these studies in identifying distinct patterns of consistent adherence, early discontinuation, and gradual decline.

Our study builds on this literature by comparing results across two regions with harmonised methodology. Comparing the results between Catalonia and Valencia cohorts, despite using the same study protocol and a common data model and programming, it is challenging to fully discern to what extent the differences in adherence observed are explained by differences in their populations, regional prescription patterns, or differences in the data sources. In Catalonia there is no direct linkage between prescription and dispensing data, whereas this linkage exists in Valencia. Although the impact of this discrepancy could not be formally quantified, both regions rely on confirmed dispensing data, which provides a comparable and valid basis for adherence estimation. This type of variability is a common challenge in multi-database studies using real-world data. Additionally, differences in socio-demographic and clinical factors may have contributed. For example, the Valencia region population had a higher mean CHA_2_DS_2_-VASc score, potentially reflecting a higher perceived risk and thus greater adherence (Hayat et al., 2023).

Although the number of classes was determined using statistical criteria (i.e., BIC), the resulting trajectories were interpretable and clinically plausible. In the Catalonia cohort, the highly adherent group identified in the 1-year or continuous analyses appeared to divide into two distinct patterns: gradual decline and gap and recovery when a more restrictive binary definition was applied over 2 years. This does not imply a lack of robustness but rather highlights behavioral nuances that emerge under different adherence metrics and follow-up durations. Such heterogeneity underscores the value of trajectory modelling in capturing adherence dynamics that would not be detected using aggregate measures alone.

Based on the identified trajectories, targeted strategies can be proposed to address specific non-adherence patterns. For instance, patients with early decline may benefit from early follow-up visits led by pharmacists or clinicians to reinforce adherence and address concerns. In contrast, patients with late decline may require long-term reinforcement strategies, such as reminder systems or periodic educational outreach. Individuals with high co-insurance could benefit from financial assistance programs or policy interventions that reduce economic barriers. Moreover, gap and recovery trajectories may reflect patients who initially struggle with adherence but can improve with appropriate support. Identifying and tailoring interventions for these subgroups could improve the real-world effectiveness of DOAC therapy. To complement these strategies, future studies should investigate the relationship between adherence trajectories and clinical outcomes to further elucidate their impact on patient health and healthcare systems.

### 4.1 Strength and limitations

A major strength of this study is the use of two population databases from the Spanish public health system, covering practically all inhabitants of the Catalonia and Valencia regions. Our study includes reliable data on clinical characteristics, including diagnoses and procedures, use of health services, prescriptions, and dispensing, and socio-demographic data at the individual level, obtained by linking several electronic databases included in the EHR. The use of both prescription and dispensing information enhances the accuracy of adherence estimation and is an advantage over studies based solely on claims data. In addition, it contributes evidence from a European setting, complementing a literature base that has focused largely on North America. A common protocol and data specifications were applied, with consistent user inclusion and exclusion criteria, operational case definitions, and common analytic procedures across the two cohorts to minimise methodological discrepancies and facilitate the comparison of results between cohort data sources. Finally, the GBTM approach provides a more nuanced understanding of adherence patterns, enabling us to identify heterogeneity in long-term adherence between patients and its evolution over the time.

There are also some limitations in our study to consider. First, potential information bias due to the differences in recording between the two population databases and the existing linkage between prescription and dispensing data. However, due to the prior homogenization performed in both databases and the sensitivity analyses applied to the common database model for the two cohorts, it is expected that the misclassification (on exposure and covariates) will be non-differential across groups of study subjects. Second, we have no information on whether the patients take the medication they fill. However, studies assessing the correlation between adherence measures from pharmacy claims and pill counts demonstrate a high level of agreement, suggesting minimal impact (Grymonpre et al., 2006). Although self-reported data may offer additional behavioural insights, it is prone to recall and social desirability bias, often leading to overestimation of adherence (Stirratt et al., 2015). In contrast, dispensing records are considered a more objective and conservative source of adherence data and are widely accepted in pharmacoepidemiologic research. Third, in our calculation of PDC, we were unable to distinguish between therapy discontinuations driven by clinical decisions (e.g., due to deterioration in liver or kidney function) and those without clinical justification. Since our data include only baseline clinical characteristics and not time-updated clinical variables, identifying such cases during follow-up was not feasible. As a result, some medically indicated discontinuations may have been inadvertently classified as non-adherence. Fourth, our analyses did not evaluate switching patterns between different types of DOAC. Therefore, the findings may not be generalizable to switchers, who may present different adherence patterns due to prior treatment experience. Future research could explore adherence trajectories specifically in this population. Finally, as we studied patients from two specific regions in Spain, the generalizability of our findings to other settings may be limited.

Despite these limitations, our findings provide a strong foundation for developing adherence-improving strategies and informing future research on the clinical implications of adherence trajectories.

## 5 Conclusion

In this retrospective cohort study, using routine clinical practice data from two regions in Spain, 3 and 5 different adherence patterns were identified at 2 years of follow-up in the Valencia and Catalonia cohorts, respectively. This approach allows the identification of distinct adherence behaviours that would not have been detected with the classical methods. Providing an opportunity to better design targeted interventions tailored to each patient group needs. Several key factors associated with poor adherence behaviours were identified, however, further research in this field is needed to better characterise the groups at higher risk of poor adherence.

## Data Availability

The data analyzed in this study is subject to the following licenses/restrictions: The data used and analysed during the current study are available from the Catalonia and Valencia region governments upon reasonable request. Data sharing is subject to ethical approval and institutional policies to protect patient confidentiality and comply with data protection regulations. Requests to access these datasets should be directed to https://aquas.gencat.cat/ca/inici.

## References

[B1] Alberts M. J. Peacock W. F. Fields L. E. Bunz T. J. Nguyen E. Milentijevic D. (2016). Association between once- and twice-daily direct oral anticoagulant adherence in nonvalvular atrial fibrillation patients and rates of ischemic stroke. Int. J. Cardiol. 215, 11–13. 10.1016/j.ijcard.2016.03.212 27104919

[B2] Alhazami M. Nik A. Dhillon S. Patel N. (2020). Medication adherence trajectories: a systematic literature review. J. Manag. Care Spec. Pharm. 26, 1138–1152. 10.18553/jmcp.2020.26.9.1138 32857646 PMC10391275

[B3] Allemann S. S. Nieuwlaat R. Jaspers F. (2019). Beyond adherence thresholds: a simulation study of the optimal classification of longitudinal adherence trajectories from medication refill histories. Front. Pharmacol. 10, 383. 10.3389/fphar.2019.00383 31105559 PMC6499004

[B4] An J. Kim J. Lee J. Cheetham T. C. Lang D. T. Fischer H. (2021). Long-term medication adherence trajectories to direct oral anticoagulants and clinical outcomes in patients with atrial fibrillation. J. Am. Heart Assoc. 10 (21), e021601. 10.1161/JAHA.121.021601 34713708 PMC8751846

[B5] Benjamin E. J. Muntner P. Alonso A. Bittencourt M. S. Callaway C. W. Carson A. P. (2019). Heart disease and stroke statistics 2019 update: a report from the American heart association. Circulation 139, e56–e528. 10.1161/CIR.0000000000000659 30700139

[B6] Chen N. Brooks M. M. Hernandez I. (2020). Latent classes of adherence to oral anticoagulation therapy among patients with a new diagnosis of atrial fibrillation. JAMA Netw. Open 3, e1921357. 10.1001/jamanetworkopen.2019.21357 32074287 PMC7081375

[B7] Dima A. L. Dediu D. Colbers A. (2017). Computation of adherence to medication and visualization of medication histories in R with adhereR. PLoS ONE 12, e0174426. 10.1371/journal.pone.0174426 28445530 PMC5405929

[B8] García-Sempere A. Orrico-Sánchez A. Muñoz-Quiles C. Hurtado I. Peiró S. Sanfélix-Gimeno G. (2020). Data resource profile: the Valencia health system integrated database (VID). Int. J. Epidemiol. 49, 740–741e. 10.1093/ije/dyz266 31977043 PMC7394961

[B9] Géric M. Piriou V. Steichen O. Bégaud B. (2017). Adherence with direct oral anticoagulants in nonvalvular atrial fibrillation new users and associated factors: a French nationwide cohort study. Pharmacoepidemiol. Drug Saf. 26 (11), 1367–1377. 10.1002/pds.4268 PMC5697683 28752560

[B10] Giner-Soriano M. Cortés J. Sabaté M. Prat-Vallverdú O. Quijada-Manuitt M. A. Morros R. (2020). The use and adherence of oral anticoagulants in primary health care in catalunya, Spain: a real-world data cohort study. Aten. Primaria 52 (8), 529–538. 10.1016/j.aprim.2020.05.016 32788057 PMC7505898

[B11] Giner-Soriano M. Prat-Vallverdú O. Cortés J. Vilaplana-Carnerero C. Morros R. (2023). Sex and gender differences in the use of oral anticoagulants for non-valvular atrial fibrillation: a population-based cohort study in primary health care in Catalonia. Front. Pharmacol. 14, 1110036. 10.3389/fphar.2023.1110036 36825151 PMC9941166

[B12] Government of Catalonia (2024). Public program of data analysis for health research and innovation in Catalonia −PADRIS. Barcelona: AQuAS. Available online at: https://aquas.gencat.cat/ca/fem/intelligencia-analitica/padris/index.html (Accessed: November 6, 2024).

[B13] Grymonpre R. Cheang M. Fraser M. Metge C. Sitar D. S. (2006). Validity of a prescription claims database to estimate medication adherence in older persons. Med. Care 44, 471–477. 10.1097/01.mlr.0000207817.32496.cb 16641666

[B14] Hayat A. Själander A. Wallvik J. (2023). Direct oral anticoagulants: patient reported adherence and minor bleedings. J. Thromb. Thrombolysis. 56, 55–64. 10.1007/s11239-023-02797-8 37119356 PMC10284977

[B15] Hernandez I. He M. Chen N. Brooks M. M. Saba S. Gellad W. F. (2019). Trajectories of oral anticoagulation adherence among medicare beneficiaries newly diagnosed with atrial fibrillation. J. Am. Heart Assoc. 8, e011427. 10.1161/JAHA.118.011427 31189392 PMC6645643

[B16] Hurtado-Navarro I. García-Sempere A. Rodríguez-Bernal C. L. Peiró S. Sanfélix-Gimeno G. Sanfélix-Gimeno G. (2018). Estimating adherence based on prescription or dispensation information: impact on thresholds and outcomes. A real-world study with atrial fibrillation patients treated with oral anticoagulants in Spain. Front. Pharmacol. 9, 1353. 10.3389/fphar.2018.01353 30559661 PMC6287024

[B17] Masnoon N. Shakib S. Kalisch-Ellett L. Caughey G. E. (2017). What is polypharmacy? A systematic review of definitions. BMC Geriatr. 17 (1), 230. 10.1186/s12877-017-0621-2 29017448 PMC5635569

[B18] Mohan A. Majd Z. Trinh T. Paranjpe R. Abughosh S. M. (2022). Group-based trajectory modeling to assess adherence to oral anticoagulants among atrial fibrillation patients with comorbidities: a retrospective study. Int. J. Clin. Pharm. 44 (4), 966–974. 10.1007/s11096-022-01417-4 35776377

[B19] Nagin D. S. (2014). Group-based trajectory modeling: an overview. Ann. Nutr. Metab. 65, 205–210. 10.1159/000360229 25413659

[B20] Nagin D. S. Odgers C. L. (2010). Group-based trajectory modeling in clinical research. Annu. Rev. Clin. Psychol. 6, 109–138. 10.1146/annurev.clinpsy.121208.131413 20192788

[B21] Nguena Nguefack H. L. Pagé M. G. Katz J. Choinière M. Vanasse A. Dorais M. (2020). Trajectory modelling techniques useful to epidemiological research: a comparative narrative review of approaches. Clin. Epidemiol. 12, 1205–1222. 10.2147/CLEP.S265287 33154677 PMC7608582

[B22] Proust-Lima C. Philipps V. Diakite A. Liquet B. (2022). Lcmm: extended mixed models using latent classes and latent processes. R. package version 2.0.0. Available online at: https://cran.r-project.org/package=lcmm.

[B23] Proust-Lima C. Philipps V. Liquet B. (2017). Estimation of extended mixed models using latent classes and latent processes: the R package lcmm. J. Stat. Softw. 78, 1–56. 10.18637/jss.v078.i02

[B24] Reilly P. A. Lehr T. Haertter S. Connolly S. J. Yusuf S. Eikelboom J. W. (2014). The effect of dabigatran plasma concentrations and patient characteristics on the frequency of ischemic stroke and major bleeding in atrial fibrillation patients: the RE-LY trial (randomized evaluation of long-term anticoagulation therapy). J. Am. Coll. Cardiol. 63, 321–328. 10.1016/j.jacc.2013.07.104 24076487

[B25] Rodríguez-Bernal C. L. Hurtado-Navarro I. Peiró S. Sanfélix-Gimeno G. Peiró S. Sanfélix-Gimeno G. (2018). Real-world adherence to oral anticoagulants in atrial fibrillation patients: a study protocol for a systematic review and meta-analysis. BMJ Open 8, e025102. 10.1136/bmjopen-2018-025102 30573490 PMC6303591

[B26] Salmasi S. Loewen P. S. Tandun R. Andrade J. G. De Vera M. A. (2020). Adherence to oral anticoagulants among patients with atrial fibrillation: a systematic review and meta-analysis. BMJ Open 10, e034778. 10.1136/bmjopen-2019-034778 32273316 PMC7245382

[B27] Salmasi S. De Vera M. A. Rahmani P. Lynd L. D. Koehoorn M. Barry A. R. (2021). Longitudinal oral anticoagulant adherence trajectories in patients with atrial fibrillation. J. Am. Coll. Cardiol. 78 (24), 2395–2404. 10.1016/j.jacc.2021.09.1370 34886959

[B28] Sherwood M. W. Douketis J. D. Patel M. R. Piccini J. P. Hellkamp A. S. Lokhnygina Y. (2014). Outcomes of temporary interruption of rivaroxaban compared with warfarin in patients with nonvalvular atrial fibrillation: results from the rivaroxaban once daily, oral, direct factor Xa inhibition compared with vitamin K antagonism for prevention of stroke and embolism trial in atrial fibrillation (ROCKET AF). Circulation 129, 1850–1859. 10.1161/CIRCULATIONAHA.113.005754 24552831 PMC4206548

[B29] Stirratt M. J. Dunbar-Jacob J. Crane H. M. Simoni J. M. Czajkowski S. Hilliard M. E. (2015). Self-report measures of medication adherence behavior: recommendations on optimal use. Transl. Behav. Med. 5 (4), 470–482. 10.1007/s13142-015-0315-2 26622919 PMC4656225

[B30] Van Gelder I. C. Rienstra M. Bunting K. V. Casado-Arroyo R. Caso V. Crijns H. J. G. M. (2024). 2024 ESC guidelines for the management of atrial fibrillation developed in collaboration with the european association for cardio-thoracic surgery (EACTS). Eur. Heart J. 45 (36), 3314–3414. 10.1093/eurheartj/ehae176 39210723

[B31] Yeaw J. Benner J. S. Walt J. G. Sian S. Smith D. B. (2009). Comparing adherence and persistence across 6 chronic medication classes. J. Manag. Care Pharm. 15, 728–740. 10.18553/jmcp.2009.15.9.728 19954264 PMC10441195

