# Correction: Real-world adherence trajectories to direct oral anticoagulants in naive patients with atrial fibrillation in Spain

**DOI:** 10.3389/fphar.2026.1843940

**Published:** 2026-04-21

**Authors:** L. M. Leguízamo-Martínez, M. E. Ballarin, I. Hurtado, G. Sanfélix-Gimeno, F. Sánchez-Sáez, M. Sabaté

**Affiliations:** 1 Department of Clinical Pharmacology, Area Medicament, Hospital Clinic of Barcelona, Barcelona, Spain; 2 Department of Clinical Pharmacology, Institut d'Investigacions Biomèdiques August Pi i Sunyer (IDIBAPS), Barcelona, Spain; 3 Department of Pharmacology, Therapeutics and Toxicology, Universitat Autònoma de Barcelona (UAB), Barcelona, Spain; 4 Clinical Pharmacology Service, Vall d'Hebron Hospital Universitari, Vall Hebron Institut de Recerca (VHIR), Barcelona, Spain; 5 Health Services Research and Pharmacoepidemiology Unit, Fundacio per al Foment de la Investigacio Sanitaria i Biomedica (FISABIO), Valencia, Spain; 6 Red de Investigación en Cronicidad, Atención Primaria y Prevención y Promoción de la Salud - RICAPPS, Barcelona, Spain; 7 Department of Applied Statistics and Operational Research, and Quality, Universitat Politècnica de València, Valencia, Spain

**Keywords:** direct oral anticoagulants (DOACs), adherence trajectories, atrial fibrillation (AF), group-based trajectory modeling (GBTM), real-world data analysis

An incorrect Funding statement was provided. The correct funder is Instituto de Salud Carlos III (ISCIII), grant number RD21/0016/0006 -Red de Investigación en Cronicidad, Atención Primaria y Promoción de la Salud (RICAPPS), Ministry of Science, Innovation and Universities, and the Recovery, Transformation and Resilience Plan through NextGenerationEU European funds.

The correct funding statement reads:

The author(s) declare that financial support was received for the research and/or publication of this article. Francisco Sánchez-Sáez was funded by Instituto de Salud Carlos III (ISCIII), grant number RD21/0016/0006 -Red de Investigación en Cronicidad, Atención Primaria y Promoción de la Salud (RICAPPS), Ministry of Science, Innovation and Universities, and the Recovery, Transformation and Resilience Plan through NextGenerationEU European funds.

The original article has been updated.

