# Supplementary material for: Real-world adherence trajectories to direct oral anticoagulants in naive patients with atrial fibrillation in Spain
Source: Front Pharmacol. 2025 Jul 31;16:1562620. doi: 10.3389/fphar.2025.1562620 (PMC12350323; doi:10.3389/fphar.2025.1562620)
Supplement: Supplementary file 1 [file Supplementaryfile2.doc]

STROBE Statement—Checklist of items that should be included in reports of ***cohort studies***

|  | Item No | Recommendation |
| --- | --- | --- |
| **Title and abstract** | 1 | (*a*) Indicate the study’s design with a commonly used term in the title or the abstract  Yes. Cohort study appears in the abstract. |
|  |  | (*b*) Provide in the abstract an informative and balanced summary of what was done and what was found  Yes. The abstract contains a balanced summary of the methods and the results. |
| Introduction | | |
| Background/rationale | 2 | Explain the scientific background and rationale for the investigation being reported  Yes. Lines 43-53 present the scientific background and the investigation motivation. |
| Objectives | 3 | State specific objectives, including any prespecified hypotheses  Yes. Lines 91-97 stablish specific objectives and prespecified hypotheses. |
| Methods | | |
| Study design | 4 | Present key elements of study design early in the paper  Yes. Lines 100-106 present the key elements of study design. |
| Setting | 5 | Describe the setting, locations, and relevant dates, including periods of recruitment, exposure, follow-up, and data collection  Yes. Lines 100-120 detail the setting. |
| Participants | 6 | Give the eligibility criteria, and the sources and methods of selection of participants. Describe methods of follow-up  Yes. Lines 122-137 explains the data sources and lines 108-120 the methods of selection of participants. |
| Variables | 7 | Clearly define all outcomes, exposures, predictors, potential confounders, and effect modifiers. Give diagnostic criteria, if applicable  Yes. In lines 139-171 this information is given. |
| Data sources/ measurement | 8* | For each variable of interest, give sources of data and details of methods of assessment (measurement). Describe comparability of assessment methods if there is more than one group  Yes, this is detailed in lines 122-146. |
| Bias | 9 | Describe any efforts to address potential sources of bias  Yes, from line 160 to line 168 are described the criteria of the trajectories selection and in the discussion section is described at lines 316-321 the efforts to avoid some sources of bias. |
| Study size | 10 | Explain how the study size was arrived at  It is not applicable in this study. |
| Quantitative variables | 11 | Explain how quantitative variables were handled in the analyses. If applicable, describe which groupings were chosen and why  Yes, this is explained in lines 139-171. |
| Statistical methods | 12 | (*a*) Describe all statistical methods, including those used to control for confounding  Yes, this is described in lines 173-187. |
|  |  | (*b*) Describe any methods used to examine subgroups and interactions  Yes, it is explained in lines 148-171. |
|  |  | (*c*) Explain how missing data were addressed  Yes. In lines 201–204, it is clarified that missing data were not problematic in this study. |
|  |  | (*d*) If applicable, explain how loss to follow-up was addressed  Yes. In lines 119–120, it is explained that patients with follow-up limitations, such as non-residents or lack of pharmaceutical coverage, were excluded from the study. In addition, the flowchart (Figure 1) explains that insufficient follow-up time was a criterion for exclusion. |
|  |  | (*e*) Describe any sensitivity analyses  Yes, in lines 105-106 there are a description of the sensitivity analysis performed. |
| Results | | |
| Participants | 13* | (a) Report numbers of individuals at each stage of study—eg numbers potentially eligible, examined for eligibility, confirmed eligible, included in the study, completing follow-up, and analysed  Yes. It is reported in the flowchart (Figure 1) as well in the results section (lines 206 and 207). |
|  |  | (b) Give reasons for non-participation at each stage  Yes. Reasons for non-participation are detailed in the flowchart (Figure 1). |
|  |  | (c) Consider use of a flow diagram  Yes. Is the Figure 1. |
| Descriptive data | 14* | (a) Give characteristics of study participants (eg demographic, clinical, social) and information on exposures and potential confounders  Yes. The Table 1 shows this information. |
|  |  | (b) Indicate number of participants with missing data for each variable of interest  No. It is not applicable in this study as it is detailed in lines 201-204. |
|  |  | (c) Summarise follow-up time (eg, average and total amount)  We are considering two specific windows of 1 and 2 years of follow-up. |
| Outcome data | 15* | Report numbers of outcome events or summary measures over time  Yes. The adherence measures are reported. |
| Main results | 16 | (*a*) Give unadjusted estimates and, if applicable, confounder-adjusted estimates and their precision (eg, 95% confidence interval). Make clear which confounders were adjusted for and why they were included  Yes, for the odds ratio (Figures 3 and 4). |
|  |  | (*b*) Report category boundaries when continuous variables were categorized  Yes. This is made for age and income. |
|  |  | (*c*) If relevant, consider translating estimates of relative risk into absolute risk for a meaningful time period  In this study, the odds ratio of different factors in relation with the trajectories has been estimated. |
| Other analyses | 17 | Report other analyses done—eg analyses of subgroups and interactions, and sensitivity analyses  Yes. The sensitivity analyses are presented as supplementary material. |
| Discussion | | |
| Key results | 18 | Summarise key results with reference to study objectives  Yes. This is done in lines 221-244. |
| Limitations | 19 | Discuss limitations of the study, taking into account sources of potential bias or imprecision. Discuss both direction and magnitude of any potential bias  Yes, from line 278 to line 334 the risk of possible biases is discussed. |
| Interpretation | 20 | Give a cautious overall interpretation of results considering objectives, limitations, multiplicity of analyses, results from similar studies, and other relevant evidence  Yes. This is found in lines 247-334. |
| Generalisability | 21 | Discuss the generalisability (external validity) of the study results  Yes. This issue is discussed in lines 309-321. |
| Other information | | |
| Funding | 22 | Give the source of funding and the role of the funders for the present study and, if applicable, for the original study on which the present article is based  Yes. In lines 349-350 the funding source is disclosed and in the lines 344-347 the author’s contributions are presented. |

*Give information separately for exposed and unexposed groups.

**Note:** An Explanation and Elaboration article discusses each checklist item and gives methodological background and published examples of transparent reporting. The STROBE checklist is best used in conjunction with this article (freely available on the Web sites of PLoS Medicine at http://www.plosmedicine.org/, Annals of Internal Medicine at http://www.annals.org/, and Epidemiology at http://www.epidem.com/). Information on the STROBE Initiative is available at http://www.strobe-statement.org.
