# Supplementary material for: Real-world adherence trajectories to direct oral anticoagulants in naive patients with atrial fibrillation in Spain
Source: Front Pharmacol. 2025 Jul 31;16:1562620. doi: 10.3389/fphar.2025.1562620 (PMC12350323; doi:10.3389/fphar.2025.1562620)

| **Table S1. International Classification of Disease, 9th and 10th edition, Clinical Modification (ICD-9-CM, ICD-10-CM) codes used to define study clinical outcomes and comorbidities.** | | |
| --- | --- | --- |
| **Clinical outcomes** | **ICD-9-CM codes** | **ICD-10-ES codes** |
| Acute Coronary Syndrome | 410.00, 410.01, 410.10, 410.11, 410.20, 410.21, 410.31, 410.40, 410.41, 410.51, 410.61, 410.70, 410.71, 410.80, 410.81, 410.90, 410.91, 411 | I20, I21 |
| GI bleeding | 456.0, 456.20, 530.21, 530.7, 530.82, 531.00, 531.01, 531.20, 531.21, 531.40, 531.41, 531.60, 531.61, 532.00, 532.01, 532.20, 532.21, 532.40, 532.41, 532.60, 532.61, 533.00, 533.01, 533.20, 533.21, 533.40, 533.41, 533.60, 533.61, 534.00, 534.01, 534.20, 534.21, 534.40, 534.41, 534.60, 534.61, 535.01, 535.11, 535.21, 535.31, 535.41, 535.51, 535.61, 537.83, 537.84, 562.02, 562.03, 562.12, 562.13, 568.81, 569.3, 569.85, 569.86, 578.0, 578.1, 578.9 | I85.01, I85.11, K22.11, K22.6, K22.8, K25.0, K25.2, K25.4, K25.6, K26.0, K26.2, K26.4, K26.6, K27.0, K27.2, K27.4, K27.6, K28.0, K28.2, K28.4, K28.6, K29.01, K29.21, K29.31, K29.41, K29.51, K29.61, K29.71, K29.81, K29.91, K31.811, K31.82, K55.21, K57.01, K57.11, K57.13, K57.21, K57.31, K57.33, K57.41, K57.51, K57.53, K57.81, K57.91, K57.93, K62.5, K63.81, K66.1, K92.0, K92.1, K92.2 |
| Intracranial haemorrhage | 430.xx, 431.xx, 432.xx, 852.0x, 852.2x, 852.4x, 853.0x | I60.x, I61.x, I62.0x, I62.1, I62.9 |
| Ischemic stroke | 433.x1, 434.x1, 436.xx | I63.x, I67.81, I67.82, I67.89 |
| Major GI bleeding | GI bleeding code + ICD-9 procedure code of blood or blood components transfusion  (99.03, 99.04, 99.05, 99.06, 99.07, 99.09) | GI bleeding code + ICD-10 prodecure code of blood or blood components transfusion  (302xxxx) |
| TIA | 435.xx | G45, I67.848 |
| **Comorbidity** | **ICD-9-CM codes** | **ICD-10 codes** |
| Alcohol | 291.xx, 303.xx, 305.0x, 357.5, 980.0 | E52.x, F10.x, G31.2, G62.1, G72.1, I42.6, K29.2, K70.x, K86.0, O35.4, T51.x, Z71.4 |
| Congestive heart failure | 398.91, 402.01, 402.11, 402.91, 404.01, 404.03, 404.11, 404.13, 404.91, 404.93, 425.4, 428.xx | I11.0, I13.0, I13.2, I42.0, I50.x |
| COPD | 496 | J44, J44.x |
| Dementia | 290.xx, 294.xx, 330.xx, 331.xx | F00.x,F01.x, F02.x, F03.x, G30.x, G31.x |
| Depression | 296.2x, 296.3x, 298.0x, 300.4x, 301.12, 311.xx | F32.x, F33.x, F34.1 |
| Diabetes | 249.xx, 250.xx, | E08.x-E11.x, E13.x |
| GI bleeding | 456.0, 456.20, 530.21, 530.7, 530.82, 531.00, 531.01, 531.20, 531.21, 531.40, 531.41, 531.60, 531.61, 532.00, 532.01, 532.20, 532.21, 532.40, 532.41, 532.60, 532.61, 533.00, 533.01, 533.20, 533.21, 533.40, 533.41, 533.60, 533.61, 534.00, 534.01, 534.20, 534.21, 534.40, 534.41, 534.60, 534.61, 535.01, 535.11, 535.21, 535.31, 535.41, 535.51, 535.61, 537.83, 537.84, 562.02, 562.03, 562.12, 562.13, 568.81, 569.3, 569.85, 569.86, 578.0, 578.1, 578.9 | I85.01, I85.11, K22.11, K22.6, K22.8, K25.0, K25.2, K25.4, K25.6, K26.0, K26.2, K26.4, K26.6, K27.0, K27.2, K27.4, K27.6, K28.0, K28.2, K28.4, K28.6, K29.01, K29.21, K29.31, K29.41, K29.51, K29.61, K29.71, K29.81, K29.91, K31.811, K31.82, K55.21, K57.01, K57.11, K57.13, K57.21, K57.31, K57.33, K57.41, K57.51, K57.53, K57.81, K57.91, K57.93, K62.5, K63.81, K66.1, K92.0, K92.1, K92.2 |
| Hypertension | 401.xx-405.xx, 437.2 | I10.x-I13.x, I15.x, I16.x, I67.4 |
| Intracranial haemorrhage | 430.xx-432.xx, 852.0x, 852.2x, 852.4x, 853.x | I60.x, I61.x, I62.0x, I62.1, I62.9, I69.x |
| Ischemic stroke | 433.x1, 434.x1, 436.xx | I63.x, I67.81, I67.82, I67.89 |
| Liver disease | 070.0, 070.2x, 070.4x, 070.6x, 070.71, 570.xx, 571.xx, 572.xx | B19.11, B15.0, B16.0, B16.2, B17.11, B19.0, B19.21, I85.x, K70.4x, K70.x, K72.x, K74.x, K75.0, K75.1, K76.6, K76.7 |
| Malignancy | 140.xx-208.xx | C00.x-C96.x |
| Other bleeding | 078.6, 246.3, 285.1, 286.5, 336.1, 360.43, 362.43, 362.81, 363.61, 363.62, 363.72, 364.41, 372.72, 376.32, 377.42, 379.23, 388.69, 423.0, 459.0, 599.7, 599.70, 599.71, 602.1, 621.4, 626.2, 626.5, 626.7, 626.8, 626.9, 640.8x, 641.1x, 641.3x, 641.8x, 641.9x, 666.1x, 666.2x, 719.1x, 782.7, 784.7, 784.8, 790.01, 866.01, 866.11, 958.2, 998.1, 998.11, 998.12 | A98.5, D62, D68.312, D68.318, D68.32, D78.0x, D78.2x, E07.89, E36.0x, E89.81x, G95.19, G97.3x, G97.5x, H05.23x, H11.3x, H21.0x, H31.30x, H31.31x, H31.41x, H35.6x, H35.73x, H43.1x, H44.81x, H47.02x, H59.1x, H59.3x, H92.2x, H95.2x, H95.4x, I31.2, I97.4x, I97.6x, J95.6x, J95.83x, K91.6x, K91.84x, L76.0x, L76.2x, M25.0x, M96.8x, N42.1, N85.7, N92.0, N92.3, N92.6, N92.x, N93.0, N93.1, N93.8, N93.9, N95.0, N99.6x, N99.82x, O20.8x, O20.x, O46.x, O72.x, R04.0, R04.1, R23.3, R31.0, R31.9, R58, R71.0, S37.019, S37.019, S37.029, S37.029, T79.2x |
| Renal disease | 403.x, 404.x, 580.x-586.x, 590.x, 753.x, V56.x | I12.x, I13.x, N00.x-N05.x, N07.x, N10, N11.x, N14.x, N15.x, N17.x-N19.x, N28.85, Q60.x, Q61.x, Z99.2 |
| TIA | 435.xx | G45, I67.848 |
| Vascular disease | 410.xx-414.xx, 440.xx, 443.9 | I20.x-I25.x, I70.x, I71.x, I73.9 |
| VTE | 415.1, 434.01, 437.6, 451.xx, 452.xx, 453.40, 453.41, 453.42, 453.5, 453.8, 453.9 | I26.x, I63.6, I67.6, I80.x, I81.x, I82.x |
| ICD-9-CM: International Classification of Diseases, 9th Revision, Clinical Modification; ICD-10-CM: International Classification of Diseases, 10th Revision, Clinical Modification; COPD: Chronic obstructive pulmonary disease, GI: gastrointestinal, TIA: Transient ischemic attack, VTE: venous and pulmonary thromboembolism. | | |

**Table S2. Population baseline characteristics at 1-year follow-up**

| **Variable** | **Category** | **Valencia (n = 19,616)** | **Catalonia (n = 22,632)** |
| --- | --- | --- | --- |
| Active Principle, n (%) | Apixaban | 6,662 (34.0%) | 7,620 (33.7%) |
|  | Dabigatran | 5,592 (28.5%) | 5,294 (23.4%) |
|  | Edoxaban | 961 (4.9%) | 2,119 (9.4%) |
|  | Rivaroxaban | 6,401 (32.6%) | 7,599 (33.6%) |
| Age, n (%) | < 65 | 4,386 (22.4%) | 5,127 (22.7%) |
|  | 65-74 | 5,772 (29.4%) | 6,115 (27.0%) |
|  | 75-84 | 6,508 (33.2%) | 7,321 (32.3%) |
|  | ≥ 85 | 2,950 (15.0%) | 4,069 (18.0%) |
|  | mean (sd) | 73.03 (11.83) | 73.49 (12.27) |
| Sex, n (%) | Man | 10,830 (55.2%) | 10,080 (44.5%) |
|  | Woman | 8,786 (44.8%) | 12,552 (55.5%) |
| Comorbidities, mean (sd) | Alcohol | 621 (3.2%) | 1,106 (4.9%) |
|  | Congestive heart failure | 3,821 (19.5%) | 2,806 (12.4%) |
|  | COPD | 1,162 (5.9%) | 2,332 (10.3%) |
|  | Dementia | 1,466 (7.5%) | 1,299 (5.7%) |
|  | Depression | 2,804 (14.3%) | 3,376 (14.9%) |
|  | Diabetes | 5,315 (27.1%) | 5,728 (25.3%) |
|  | Gastrointestinal bleeding | 995 (5.1%) | 1,313 (5.8%) |
|  | Hypertension | 14,748 (75.2%) | 15,374 (67.9%) |
|  | Intracranial Haemorrhage | 459 (2.3%) | 345 (1.5%) |
|  | Ischemic stroke | 2,513 (12.8%) | 2,600 (11.5%) |
|  | Liver disease | 697 (3.6%) | 225 (1.0%) |
|  | Malignancy | 2,583 (13.2%) | 4,147 (18.3%) |
|  | Other bleeding | 2,683 (13.7%) | 2,214 (9.8%) |
|  | Renal disease | 2,492 (12.7%) | 3,290 (14.5%) |
|  | TIA | 940 (4.8%) | 1,131 (5.0%) |
|  | Vascular disease | 4,535 (23.1%) | 4,516 (20.0%) |
|  | VTE | 1,500 (7.6%) | 1,352 (6.0%) |
| Scores, mean (sd) | CHADS₂ | 2.03 (1.38) | 1.88 (1.36) |
|  | CHA₂DS₂-VASc | 3.49 (1.85) | 2.85 (1.71) |
|  | HAS-BLED | 2.61 (1.26) | 2.48 (1.30) |
| CHA₂DS₂-VASc Categories, n (%) | 0-1 | 3,006 (15.3%) | 5,205 (23.0%) |
|  | 2 | 3,103 (15.8%) | 4,390 (19.4%) |
|  | 3 | 3,811 (19.4%) | 5,365 (23.7%) |
|  | 4 | 3,946 (20.1%) | 3,754 (16.6%) |
|  | ≥5 | 5,750 (29.3%) | 3,918 (17.3%) |
| HAS-BLED Categories, n (%) | 0-1 | 3,664 (18.7%) | 5,172 (22.9%) |
|  | 2 | 5,386 (27.5%) | 6,382 (28.2%) |
|  | 3 | 6,000 (30.6%) | 6,190 (27.4%) |
|  | ≥4 | 4,566 (23.3%) | 4,888 (21.6%) |
| Treatment in the 3 Months Previous to the Index Date, n (%) | APT | 8,703 (44.4%) | 9,678 (42.8%) |
|  | NSAID | 2,252 (11.5%) | 2,594 (11.5%) |
|  | Coxib | 816 (4.2%) | 230 (1.0%) |
|  | anti-arrhythmic | 3,593 (18.3%) | 3,489 (15.4%) |
| Polypharmacy, n (%) | mean (sd) | 7.78 (4.05) | 5.24 (3.04) |
|  | ≥ 5 | 15,359 (78.3%) | 12,455 (55.0%) |
| Coinsurance^*^ | low (0%-10%) | 16,228 (82.7%) | 19,656 (86.9%) |
|  | high (40%-60%) | 3,388 (17.3%) | 2,976 (13.1%) |
| ^*^Coinsurance is expressed as a percentage of the total cost of prescriptions to be paid. AF = atrial Fibrillation; COPD = chronic obstructive pulmonary disease; VTE = venous thromboembolism; APT = antiplatelet therapy; NSAID = non-steroidal anti-inflammatory drugs. | | | |

**Supplemental Figure S1. Temporal evolution of incorporation for both cohorts**

**
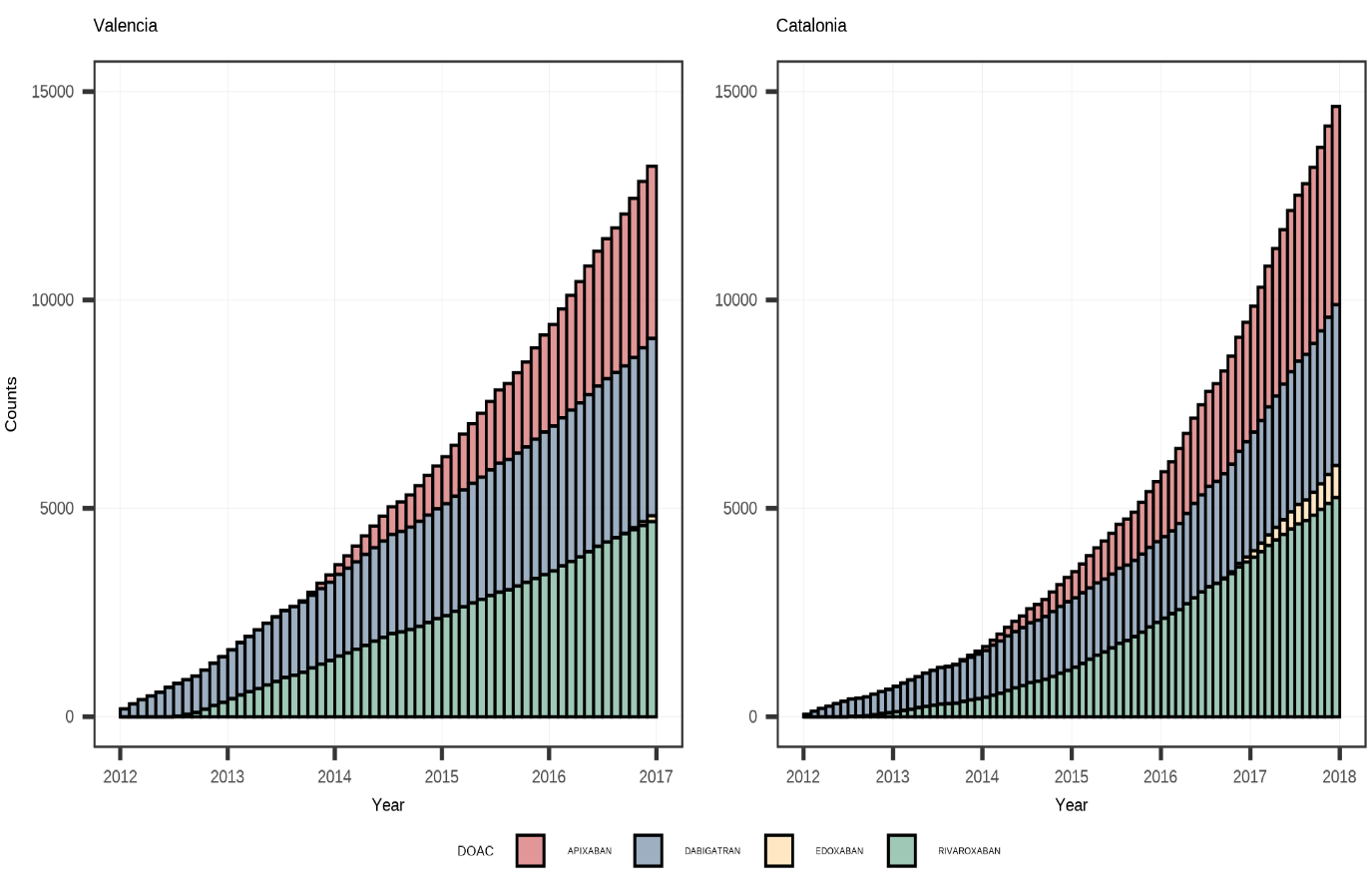
**

**Supplemental Figure S2. Adherence trajectories at 1 year follow-up (Binary adherence).**


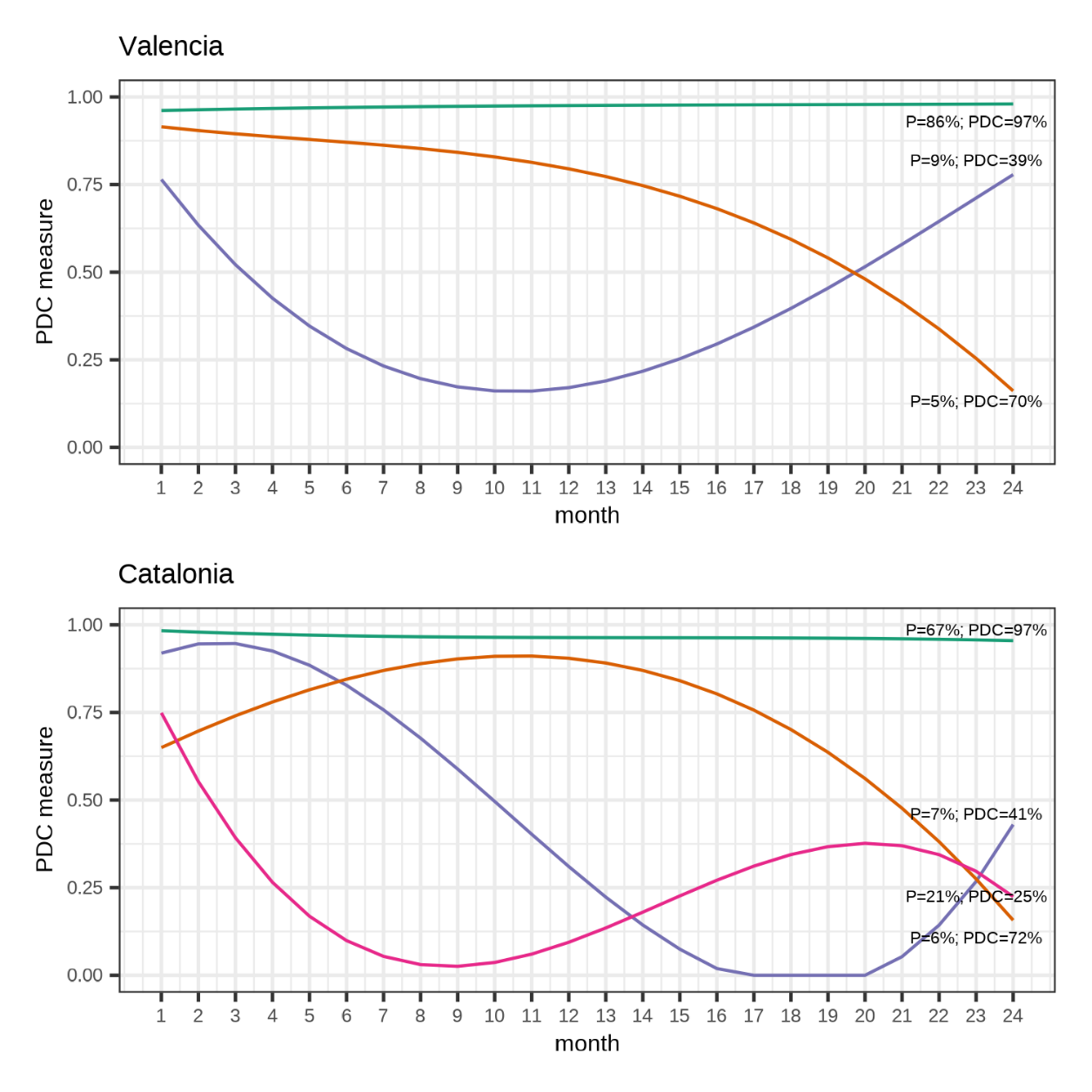


**Supplemental Figure S3. Adherence trajectories at 2-years follow-up (Continuous adherence outcome).**

**
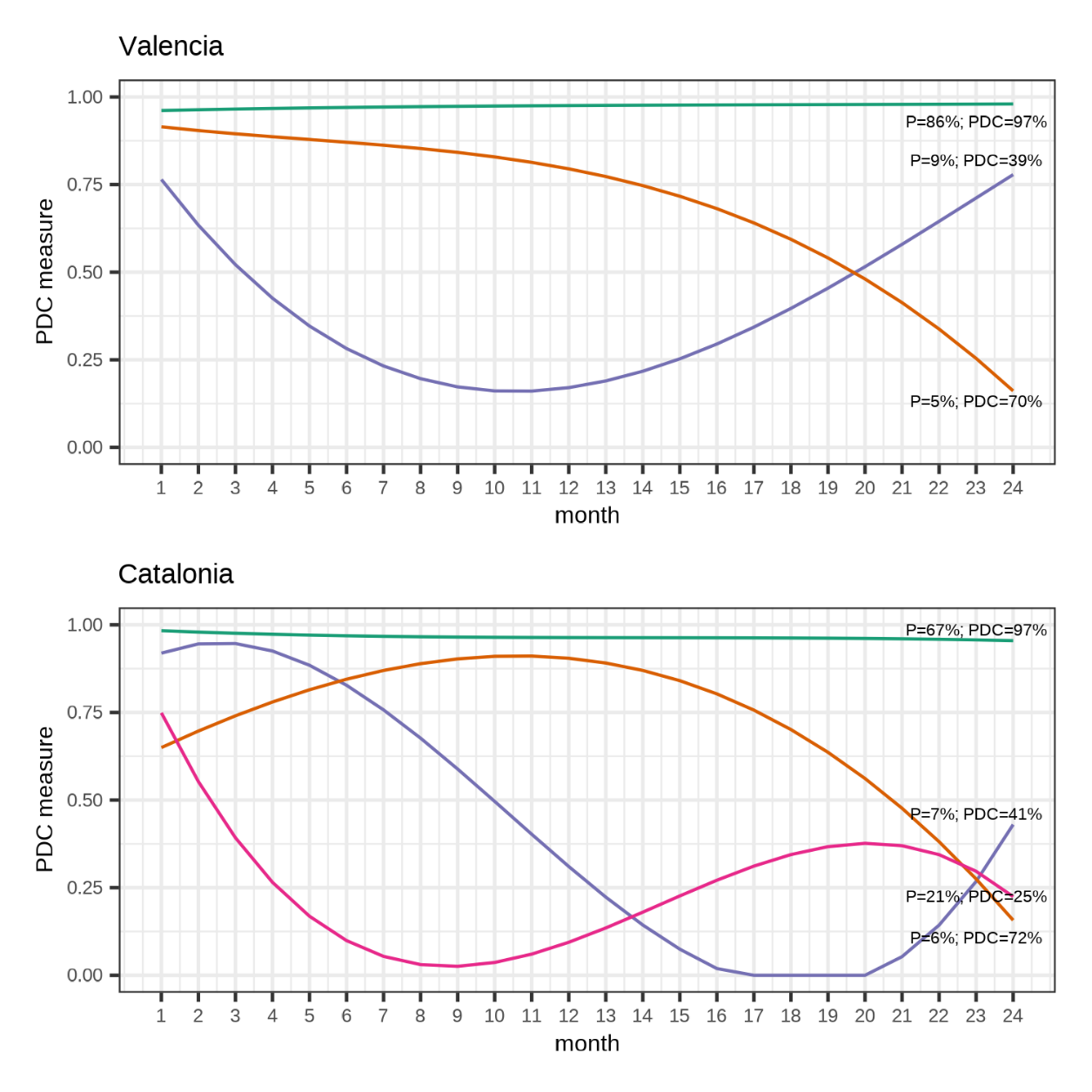
**

**Supplemental Figure S4. Adherence trajectories at 1-year follow-up (Continuous adherence outcome).**


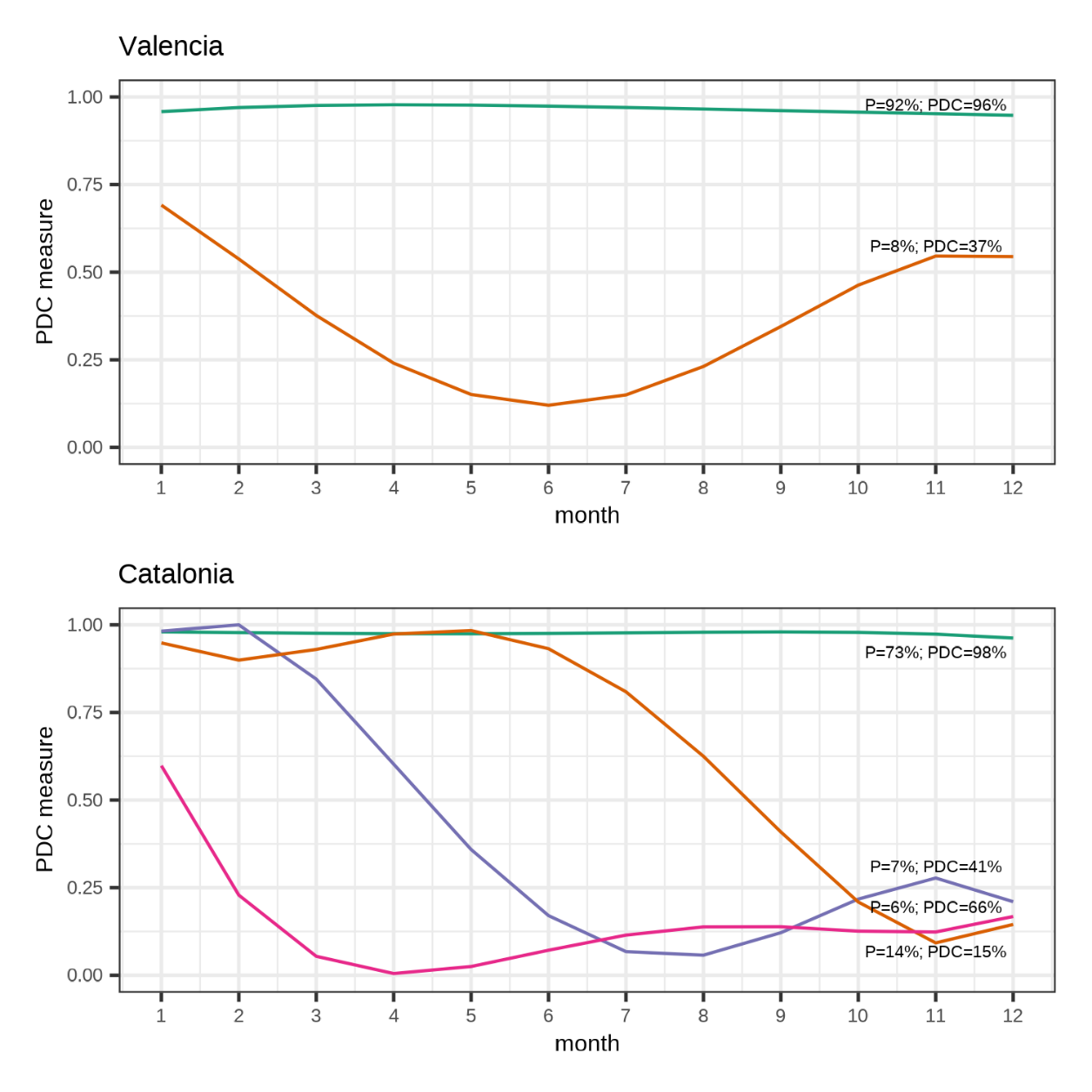


Figure S5a. Factors associated with Catalonia adherence trajectories at 1-year follow-up.


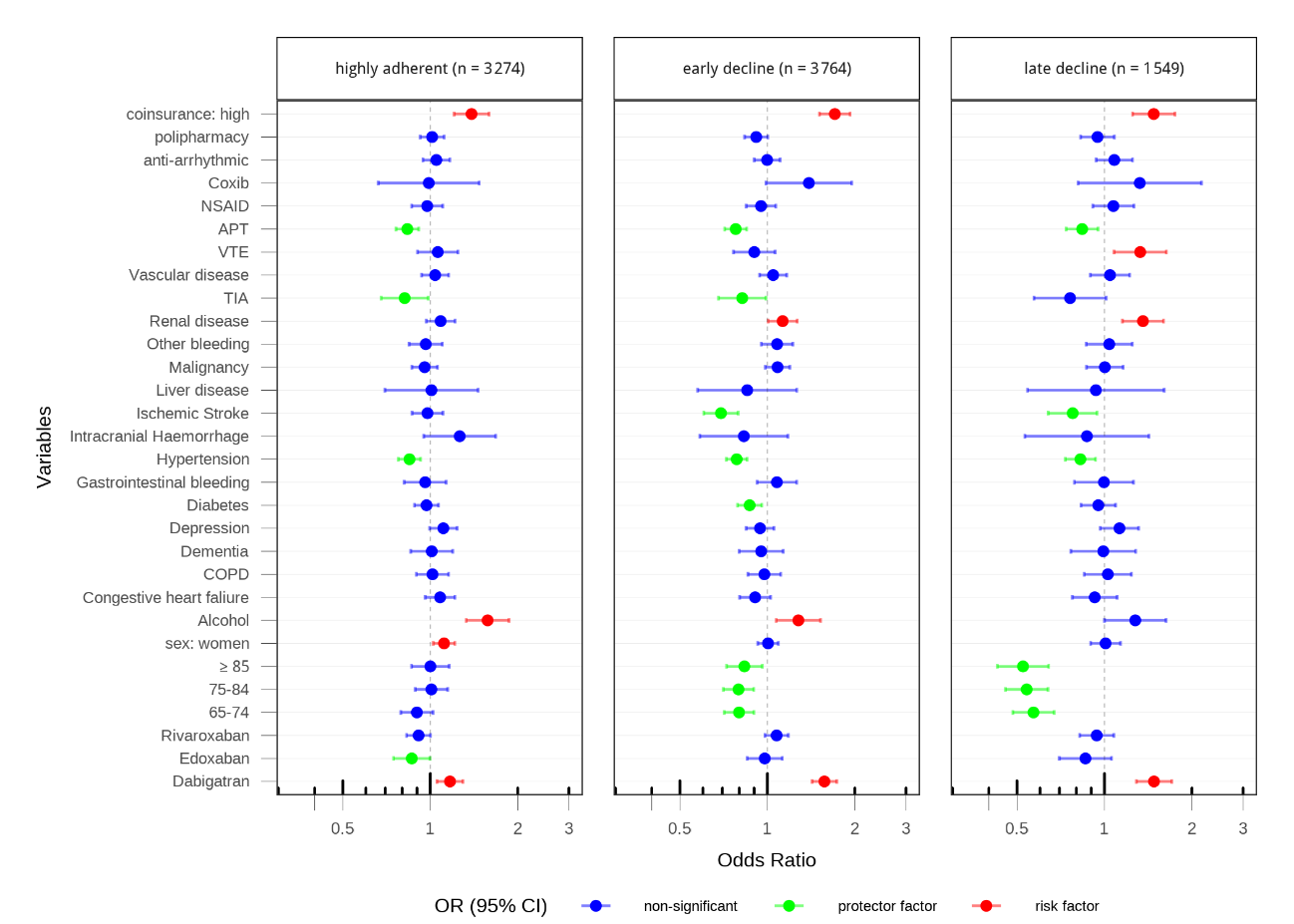


Figure S5b. Factors associated with Valencia adherence trajectories at 1-year follow-up


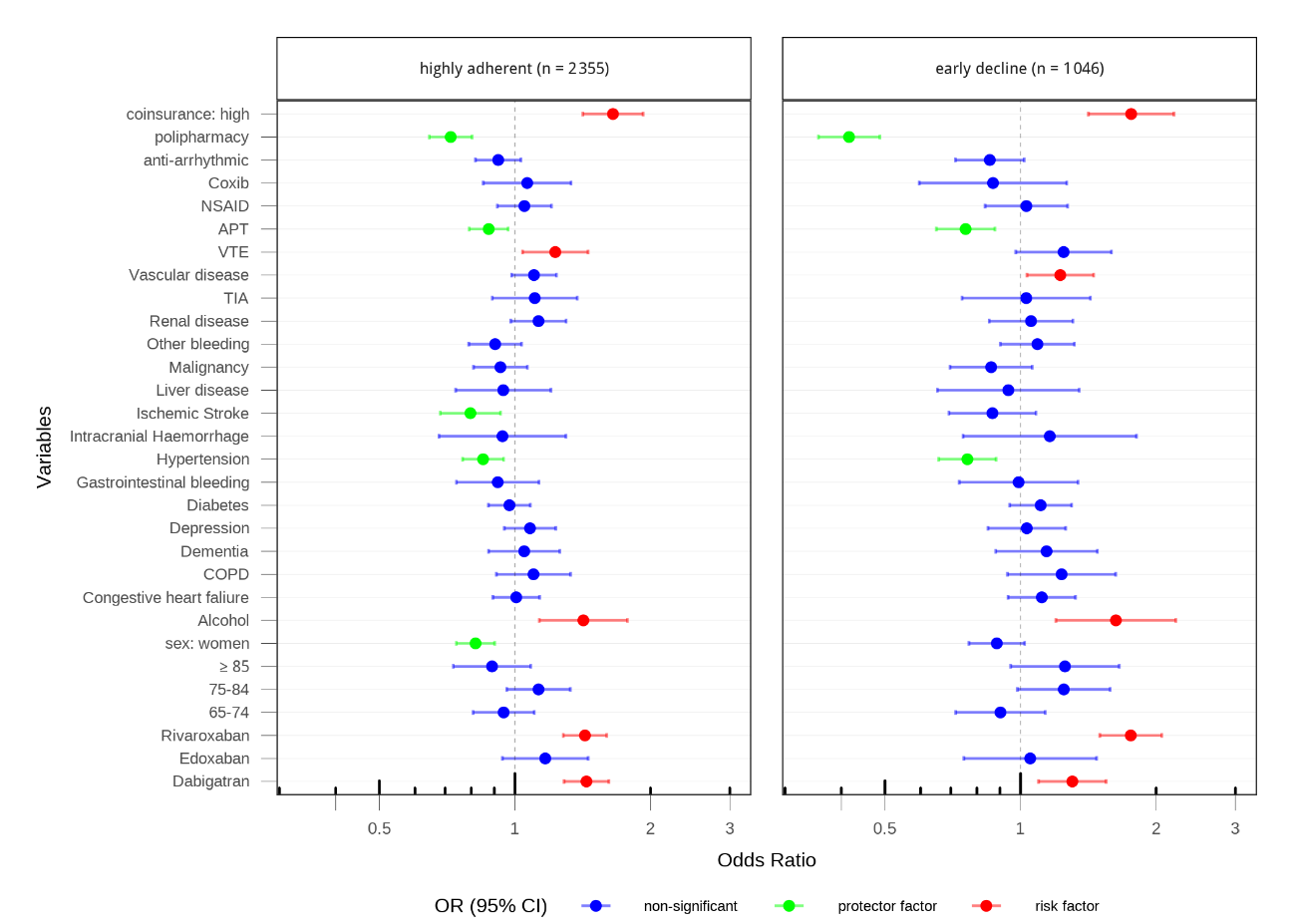

Supplement: Supplementary file 2 [file Supplementaryfile1.docx]
